# Visiting parents in times of COVID-19: The impact of parent-adult child contacts on the psychological health of the elderly

**DOI:** 10.1016/j.ehb.2022.101152

**Published:** 2022-08

**Authors:** Agar Brugiavini, Cinzia Di Novi, Cristina Elisa Orso

**Affiliations:** a Department of Economics, Ca’ Foscari University of Venice, Italy; b European Commission, Joint Research Centre (JRC), Ispra, Italy; c Department of Law, Economics, and Cultures, Insubria University, Como, Italy

**Keywords:** COVID-19, Stay-at-home order, Parent–adult child relationship, Disruption, Mental health

## Abstract

Using the 8th wave of the SHARE and the SHARE Corona Survey, we investigated whether the disruption of parent–adult child contacts due to social distancing restrictions increased the symptoms of depression among old age individuals during the first wave of the COVID-19 pandemic. We model the relationship between the disruption of parent–adult child contacts and the mental health of the elderly using a recursive simultaneous equation model for binary variables. Our findings show that the likelihood of disruption of parent–adult child contacts was higher with adult children who do not live with or close to their parents (i.e., in the same household or in the same building) for whom contact disruption increases about 15 %. The duration of restrictions to movement and lockdowns also has a positive and significant effect on parent-child contact disruption: an additional week of lockdown significantly increases the probability of parent-child contact disruption, by about 1.5 %. The interventions deemed essential to reduce the spread of the pandemic, such as the “stay-at-home” order, necessarily disrupted personal parent–child contacts and the social processes that facilitate psychological well-being, increasing the probability of suffering from a deepening depressed mood by about 17 % for elderly parents.

## Highlights

• We explore whether the disruption of the parent–child contacts, because of lockdown, increases depression in older persons.
• We use a joint model of contacts disruption and mental health to takes into account for unobserved heterogeneity.
• Our evidence reflects that parent-adult child contacts disruption due to COVID‐19’s worsens mental health of older people.
• Our study provides additional insights into the psychological status and strain on older during the COVID-19 outbreak.

## 1 Introduction

The worldwide crisis due to the COVID-19 pandemic is having an impact on almost every aspect of our society. At the beginning of the pandemic, when no medicines or vaccines were available, countries relied on other types of intervention, including social distancing: isolation, quarantine, travel restrictions and the closure of schools, universities, workplaces and public spaces. Even though social distancing has reduced the rate of infection, naturally it has come at the cost of an economic crisis and forgoing the benefits of physical and social contacts (Soucy et al., 2020; Fang et al., 2020). Lockdown might have exacerbated individuals’ pre-existing mental health issues and negatively affected their well-being (due to increased anxiety, stress and other negative feelings and concerns about the practical implications of the response to the pandemic, including financial difficulties) (Giuntella et al., 2021, Mata et al., 2021; White & Van der Boor, 2020; WHO, 2020; Tucci et al., 2017).

Although, at the time of writing, the full effects of COVID-19 and the associated crisis are yet to be seen, it is expected that they will not be uniform. Vulnerable and disadvantaged groups will be impacted more severely. Older adults, in particular, have a higher risk of infection from COVID-19: and are more likely already to be suffering from multiple chronic diseases such as cardiovascular disease, diabetes, or respiratory illness, increasing the risk of severe COVID-19 consequences, including death (Flaherty et al., 2020; Steptoe et al., 2021).

The balance between age-related disorders and good health during the lockdown suffered immense pressure. On average, older adults have smaller social networks and lower rates of social contacts than middle-aged and younger adults (Cornwell, 2011). Life-course transitions such as retirement, declining economic resources, widowhood but also impaired mobility and cognitive decline may lead the elderly to be less engaged in community activities reducing overall rates of contact and increasing the risk of isolation (Donnelly and Hinterlong, 2010, Cornwell and Laumann, 2015). During the pandemic, social distancing measures, which were often necessary to protect older adults from the risk of coronavirus, further altered the social connectedness of the elderly: in-person contact with non-resident family and friends have been severely curtailed given the very high rates of COVID-19 morbidity/mortality in this population (Rochon et al., 2020). Older adults are particularly susceptible to social isolation since they are in general also less familiar with communications technology, essential to overcome the lack of in-person social contacts during the pandemic (Rolandi et al., 2020). However, the elderly, especially those suffering a cognitive decline or dementia, need emotional support through informal networks. Isolation may have created a new set of challenges affecting pre-existing health conditions, including mental health (Bu et al., 2020).

Historically, in Europe, family members and in particular (non-cohabiting) adult offspring, have provided most of the informal care to those in later life; much lower proportions of older people receive regular help from friends or neighbors (Brenna and Di Novi, 2016, Di Novi et al., 2015). The support of sons and daughters continues to play a substantial role in the total care provided, especially among frail older people who depend on youngsters for their daily needs. Contacts between adult children and elderly parents and the informal care provided by adult children provide important support for the elderly and are a valuable substitute for, and complement of, formal care particularly in welfare states where there are strong family bonds (Van den Berg et al., 2004; Van den Berg et al., 2005). According to the existing literature the support provided by offspring positively affects the physical and emotional well-being of parents throughout their lives and is especially crucial in later life (Albertini and Mencarini, 2014, Litwin and Stoeckel, 2013; Litwin et al., 2015; Litwin and Shiovitz-Ezra, 2011).

Prior research on parent–child relationships and the well-being of older parents has mostly focused on the frequency of contacts, assuming that the more numerous the contacts, the greater the parent’s well-being (Mancini and Blieszner, 1989; Tomassini et al., 2004). However, the COVID-19 pandemic and the consequent self‐isolation, has led to a disruption of parent–adult child contacts. Indeed, in the recent literature on symptoms of depression in older persons related to the COVID-19 pandemic, one neglected topic is the influence of the disruption of the parent–adult child relationship on the psychological and emotional well-being of the elderly parent. Using data from the 8th wave of the Survey of Health, Ageing and Retirement in Europe (SHARE) until its suspension in March 2020 and the SHARE Corona Survey fielded from June to August 2020, this study aims to fill this gap providing additional insights into the psychological status of, and strain on, older parents during the COVID-19 outbreak and contributing to the very limited, but growing body of research on the negative association between social isolation and the psychological well-being of the elderly.

The remainder of the paper is organized as follows. Section 2 sets out the data, Section 3 describes the empirical strategy, while the results are presented and discussed in Section 4. The concluding remarks are in Section 5.

## 2 Data

This study makes use of individual-level data drawn from the 8th wave of the Survey of SHARE and the SHARE Corona Survey. The 8th wave of SHARE is a regular wave collecting information on the health, demographic and socio-economic status of individuals who are 50 years old or over. The interviews took place between October 2019 and March 2020. A sub-sample of SHARE panel respondents was interviewed from June to August 2020, via a Computer Assisted Telephone Interview (CATI), partly to collect a set of basic information as in the regular SHARE questionnaire, and partly to elicit information on life circumstances in the presence of COVID-19. The data collected with the latter questionnaire provide a detailed picture of how older adults were coping with the health-related and socio-economic impact of COVID-19 (Scherpenzeel et al., 2020). It also included the most important life domains for the target population and specific questions about the COVID-19 infection and life changes during the lockdown i.e. physical health (general health before and after the COVID-19 outbreak, infections and COVID-19 related symptoms); mental health (anxiety, depression, sleeping problems and loneliness before and after the COVID-19 outbreak); health behavior (social distancing, mask wearing etc.); SARS-CoV-2 testing and hospitalization; any medical treatment missed; satisfaction with treatment; changes in work and the patient’s economic situation; social networks (changes in personal contacts with family and friends, help given and received, personal care given and received).

Our sample consists of 15,508 individuals living in 24 European countries plus Israel.

This paper focuses specifically on individuals aged 65 and over. The COVID-19 pandemic has taken a heavy toll on their physical as well as mental health. The restrictive measures taken by governments (social distancing and isolation) to prevent the spread of the infection, have often resulted in social isolation and loneliness, to which older adults are likely more vulnerable because of their functional dependency, which in turn may have increased their depression and cognitive dysfunction with significant consequences for their psychological well-being (Banerjee, 2020).

## 3 Empirical strategy

### 3.1 Main variables

The aim of our paper is to investigate the effect of an unexpected disruption of parent–child contacts due to COVID-19 in terms of the mental health of elderly parents. We constructed a binary indicator of a worsened depressed mood based on the SHARE Corona Survey. Respondents were asked to report whether, in the month before the interview, they felt sad or depressed. If the answer was “yes”, they were also asked to report whether they felt sad or depressed “more”, “less”, or “about the same” compared to the period before the COVID-19 outbreak. Based on the answers, we created a dummy variable with value 1 if respondents, who said they felt sad or depressed, also said they were more depressed than before the COVID-19 pandemic outbreak, and 0 otherwise (less or about the same).,^1^^.^^2^

In order to measure the potential disruption of the parent-child relationship due to the pandemic, we created a binary variable taking into account the variation in the frequency of parent-child contacts in the periods before and during the first wave of the COVID-19 outbreak. Specifically, the 8th wave of SHARE includes a module on respondents’ personal social networks. Each respondent can name up to seven people considered confidants. The social network module also gathers information on a respondent’s relationship with these confidants (children, relatives, friends and neighbors) as well as additional characteristics for each social network member (gender, degree of kinship, network proximity). Using information drawn from the social network module, we were able to identify children considered by respondents to be “confidants”. In addition, we established whether respondents had regular contacts with them before the COVID-19 outbreak (either in-person, by phone, email or any other electronic means). By “regular contacts” we mean either (i) daily; (ii) several times a week, (iii) about once a week (against about every two weeks, less than once a month and never). Using the information in the SHARE Corona Survey, we were also able to elicit the frequency of respondents’ contacts with their “confidant” children during the COVID-19 outbreak.

**Fig. 1:**
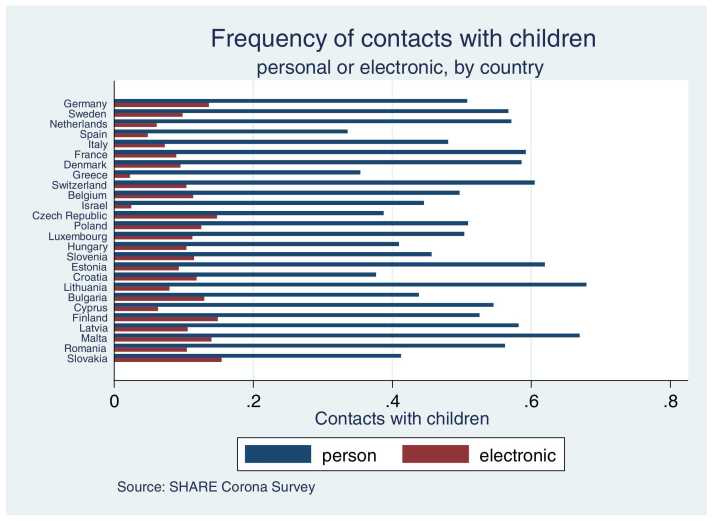
Individuals aged 65 + reporting personal or electronic contacts with offspring “less often” or “never” since the outbreak.

In order to generate a variable to accurately measure the disruption of parent-children contacts, we compared the respondents’ answers to the questions concerning the frequency of in-person and electronic contacts, as in the social network module of the 8th wave of SHARE with the answers reported in the SHARE Corona Survey. We constructed a binary variable with value one if those reporting regular contacts with confidant children living outside the household (in-person, by phone, email or any other electronic means) before the outbreak also reported contacts with them “less often” or “never” during the outbreak. Unfortunately, while the SHARE Covid Survey enables different forms of contact with social network members to be distinguished—e.g., in-person, by phone or mail, email or any other electronic means, the 8th wave of SHARE does not distinguish between in-person and electronic contacts with people who live outside the household (children, parents, relatives, friends and neighbors). We assumed that the negative trend in the frequency of contacts with children was mainly due to the disruption of face-to-face contacts. Indeed, the restrictions many countries adopted during the first wave of the pandemic meant that people had to stay home and avoid all contacts with non-household members. This is confirmed by our data. In Appendix, Fig. 1 shows the fraction of individuals aged 65 + in each country who reported personal or electronic contacts with offspring “less often” or “never” since the outbreak. The majority of respondents who stated “less often” or “never” indicated face-to-face contacts. On average, about 50.7 % of our sample had personal contacts with children “less often” or “never” since the outbreak, against about 10.2 % for electronic contacts.^3^

### 3.2 Econometric model: the Recursive bivariate probit

From the methodological point of view, it should be noted that associating the disruption of parent–children contacts with the psychological well-being of the elderly may be complicated by the presence of endogeneity. The disruption of parent–children contacts associated with the pandemic might have undermined older adults’ mental health which, in turn, might have simultaneously influenced parental access to the informal support of their offspring (Cacioppo et al., 2006, Holt-Lunstad et al., 2010). In order to take this potential simultaneous relationship into account, we adopted a recursive bivariate probit model. The recursive structure of the bivariate probit model is built on a first structural form equation determining the probability of a deterioration in mental health conditions (*y*_*1i*_ in the Eq. (1)) and a second reduced form equation for the potentially endogenous dummy measuring the disruption of the parent–adult child relationship (*y*_*2i*_ in the Eq. (1)). In the probit model used to predict a deterioration of mental health, among the dependent variables, we included the indicator of disruption of the parent–adult child relationship (*y*_*2i*_). Thus:

(1) $${\;y}_{1i}^{*}=\beta_{1}^{\prime }x_{1i}+\varepsilon_{1i}=\delta_{2}y_{2i}+\alpha^{\prime }z_{i}+\varepsilon_{1i}$$

$$y_{2i}^{*}=\beta_{2}^{\prime }x_{2i}+\varepsilon_{2i}$$

where $z_{i}$ and $x_{2i}$ are vectors of exogenous variables, α and $\beta_{2}$ are parameter vectors, $\delta_{2}$is a scalar parameter. $\varepsilon_{1i}$ and $\varepsilon_{2i}$ are the error terms distributed as bivariate normal, each with a mean zero and a variance covariance matrix ∑. ∑has values of 1 on the leading diagonal and correlations._._$\rho_{21}$ on off-diagonal elements. In the above setting, the exogeneity condition is stated in terms of the correlation coefficient, which can be interpreted as the correlation between the unobservable explanatory variables of the two equations. The equations in (1) can be estimated separately as single probit models only in the case of independent error terms, i.e., the correlation coefficient is not significantly different from zero.^4^

#### 3.2.1 Identification Strategy

Conventionally, the identification of a recursive bivariate probit model has been based on exclusion restrictions to obtain a more robust identification of the parameters. Maddala (1983, p. 123) suggests that at least one of the reduced-form exogenous variables be not included in the structural equations as explanatory variables. However, recently, Wilde (2000) demonstrated that identification is achieved even if the same regressors appear in both equations provided there is sufficient variation in the data. Specifically, first, following Maddala’s approach, we imposed exclusion restrictions, then following Wilde (2000) on identification of multiple equation probit models, we re-estimated a bivariate model where each equation has the same regressor matrix (see the Appendix Section 1 A).^5^

According to Maddala (1983) to achieve identification, the $z_{i}$ equation should not include all the variables included in the $x_{2i}$ equation. For the reduced form (i.e., the disruption equation for parent-child contacts), we included a variable which was assumed to directly affect the disruption of parent–adult child contacts and only indirectly the probability of a deterioration in mental health. Specifically, to adopt an appropriate instrument to predict the reduced form equation, we used information from the social network module included in wave 8 of the SHARE survey (where the data were collected before the COVID-19 outbreak): for children named by respondents as confidants, SHARE provides additional information including where they live. Specifically, respondents were asked the following question: *Where does “name of confidant” live? 1. In the same household; 2. In the same building; 3. Less than 1 kilometre away; 4. Between 1 and 5 kilometres away; 5. Between 5 and 25 kilometres away; 6. Between 25 and 100 kilometres away; 7. Between 100 and 500 kilometres away; 8. More than 500 kilometres away.* Based on this information, we created a dummy variable with value 1 if “confidant” children did not live in the same household or in the same building, and 0 otherwise.

Along with information on non-cohabiting vs cohabiting offspring, we considered the implementation of lockdown in European countries, and data were taken from the open-access database of the European Centre for Disease Prevention and Control (European Centre for Disease Prevention and Control Data, 2020).^6^ At the beginning of March 2020, many European countries in response to the COVID-19 pandemic imposed a nationwide lockdown (aka “shelter-in-place” or “stay-at-home”) to reduce contagion and alleviate pressure on healthcare systems. Lockdowns limited the free circulation of people and prohibited “non-essential” services and activities leading to one of the largest enforced isolation periods in human history (Di Novi et al., 2022). Specifically, we constructed an indicator of duration of the lockdown restrictions measured as the number of weeks since the beginning of the lockdown in each country in the study. The length of the “isolation period” differs between countries and may have had varying degrees of impact. For instance, in Spain, Italy and France the lockdown lasted about 8 weeks, while in other countries such as Poland it was for about 4 weeks. Parent-child non-cohabitation and the duration of lockdown restrictions were assumed to be exogenous instruments for the disruption of parent-adult child relationships.

COVID-19 restrictions to movement and the fear of infection rapidly and dramatically changed people’s interactions. Technology was adapted to mitigate the disruption to the social network, offering individuals digital alternatives to the face-to-face contacts generally rendered impossible by the Covid crisis (Newman and Zainal, 2020). The dependent variable of the reduced form equation includes electronic contacts with children. Hence, included among the regressors is an indicator of general and regular Internet use in everyday life, as per the eighth wave of SHARE, as a measure of older users’ ability to harness the Internet to cope with pandemic-induced social network disruption. The variable was constructed according to the question: “*During the last 7 days, have you used the Internet, for e-mailing, searching for information, making purchases, or for any other purpose at least once*?” Again, we constructed a binary variable with value one if respondents answered yes, and zero otherwise.^7^

#### 3.2.2 Additional independent variables

In our model, we also control for a rich set of individuals’ demographic and socio-economic characteristics and health variables. For demographics, we included the respondent’s sex (0: male, 1: female) and age. The International standard classification of education (Isced) was used to classify the education variable. Three levels of education were considered: (1) low education (no educational certificates or primary school certificate or lower secondary education); (2) medium education (upper secondary education or high school graduation); (3) high education (university degree or postgraduate). Marital status was categorized as ‘living with a spouse or a partner in the same household’ and ‘living as single’.

We also included an indicator of current financial distress to proxy the household’s ability to make ends meet. Participants were asked to think about the household’s total monthly income and rate the degree to which they felt able to make ends meet: with great difficulty, with some difficulty, fairly easily or easily. This information was treated as a dummy variable with value one if respondents reported “with great difficulty” or “some difficulty” and zero otherwise. While in a long-term view one may argue that financial distress is also endogenous, in the short time period the survey captures it is likely that the economic impact of the lockdown measures did have an immediate effect on the main variables of interest.

In order to capture “needs” unrelated to the pandemic itself and the associated lockdown, we also included information on respondents’ health status before the outbreak (between October 2019 and February 2020). The health-related variables included the number of self-reported chronic diseases (high blood pressure; high cholesterol; stroke; diabetes; chronic lung disease; asthma; arthritis, osteoporosis; cancer; peptic ulcer; Parkinson’s disease; cataracts; hip fracture; or other conditions); the number of self-reported problems with mobility (walking 100 m; sitting for about two hours; getting up from a chair after sitting for long periods; climbing several flights of stairs without resting; climbing one flight of stairs without resting; stooping, kneeling, or crouching; reaching or extending the arms above shoulder level; pulling or pushing large objects such as a living room chair; lifting or carrying weights over 5 kilos; or picking up a small coin from a table) and an indicator of cognitive functions. Following Bonsang et al. (2012), in our empirical analysis, we focused on one key cognitive domain: memory recall (episodic memory). The test relies on the immediate and delayed recall of a 10-word list. The interviewer reads out 10 common words (e.g., book, child, hotel, etc.). In the immediate recall, participants are asked to recall as many words as possible in one minute, immediately after hearing them. In the delayed recall, participants are asked to recall as many words in one minute, after several other interview questions. Each word correctly recalled scores 1 point. Finally, the episodic memory score is calculated by adding up the number of target words recalled immediately and the number of target words recalled after the delay. Thus, the score ranges between 0 and 20 with a high score indicating good cognitive function (Bonsang et al., 2012, Grasshoff et al., 2021).

In addition to social isolation, the local virus spread might also be a key factor in determining mental health issues and the disruption of social network contacts during a lockdown. Therefore, we considered a set of variables related to the COVID-19 experience, including a variable that provides information on the spread of COVID-19 among respondents’ contacts. This dummy indicator has value one if anyone close to a respondent (i) had suffered from the Coronavirus; (ii) was hospitalized due to the infection; (iii) died after being affected by the Coronavirus, and 0 otherwise. We also introduced a measure that indicates whether the individual was directly affected by COVID-19, using a set of questions to establish if a respondent (i) had experienced symptoms, (ii) had been tested for COVID-19, and/or (iii) had been hospitalized (Bergmann and Wagner, 2021). According to recent studies, symptoms indicating COVID-19 are associated with higher rates of anxiety and depression (Rajkumar, 2020, Le and Nguyen, 2021). We also included an indicator of personal distance behavior, expected to influence the disruption of contacts with social networks (including adult children) and mental health.

Finally, in the empirical model, we included an indicator of regular contacts (either in-person or by phone, email, or any other electronic means) with a relative/non-relative during the outbreak and country dummies to control for country fixed effect differences.

Table 1 sets out a full description of the variables used in the model.

**Table 1:** Variables.

Variable name	Description	Data Sources
Deepening depressed mood	1 if sadder or depressed since the outbreak, 0 otherwise	SHARE Corona Survey
Greater Anxiety	1 if more nervous since the outbreak, 0 otherwise	SHARE Corona Survey
Greater Loneliness	1 if lonelier since the outbreak, 0 otherwise	SHARE Corona Survey
Disruption of Parent-child contacts	1 if disruption in the contacts with children was experienced, 0 otherwise	SHARE Wave 8/SHARE Corona Survey
Age	Continuous variable	SHARE Corona Survey
Female	1 if female, 0 otherwise	SHARE Corona Survey
Married	1 if married, 0 otherwise	SHARE Corona Survey + previous waves*
Low education level	1 if poorly educated, 0 otherwise	SHARE Corona Survey + previous waves
Medium education level	1 if medium educated, 0 otherwise	SHARE Corona Survey + previous waves
High education level	1 if highly educated, 0 otherwise	SHARE Corona Survey + previous waves
Retired	1 if retired, 0 otherwise	SHARE Corona Survey + previous waves
Ends not meeting	1 if able to make ends meet with great difficulty or with some difficulty; 0 otherwise	SHARE Wave 8
# Chronic conditions	Number of self-reported chronic conditions	SHARE Wave 8
#Mobility limitations	Number of self-reported problems with mobility before the outbreak	SHARE Wave 8
Episodic memory score	Score (from 0 to 20) measuring episodic memory - a higher score corresponds to better cognitive functions	SHARE Wave 8
Directly affected Covid-19	1 with symptoms and/or having been tested for COVID-19, and/or having been hospitalized; 0 otherwise	SHARE Corona Survey
Other people with Covid 19	1 if anyone close had suffered from the Coronavirus, and/or was hospitalized due to the infection, and/or died after being affected by the Coronavirus; 0 otherwise	SHARE Corona Survey
Physical distance	1 if distance was kept “always” or “often” when outside the home; 0 otherwise	SHARE Corona Survey
Any contact with others	1 if with electronic or in-person contacts with relatives, friends, and neighbors; 0 otherwise	SHARE Corona Survey
Stay-at-Home restrictions (weeks)	Number of weeks the country of residence adopted lockdown restrictions, 0 otherwise	European Centre for Disease Prevention and Control (2020)
Non-cohabitation	1 if “confidant” children did not live in the older parents’ household or in the same building, 0 otherwise.	
	European Centre for Disease Prevention and Control (2020)	

N.B.: * information about education and marital status were retrieved from previous waves for longitudinal respondents.

## 4 Results and discussion

Table 2 provides a simple descriptive analysis, presenting sample means and standard deviations for the variables used in the model (55% female; mean age: 74 years old). Notably, according to our definition of depression, around 15% of the sample reported feeling more depressed since the Covid-19 outbreak compared to the pre-Covid period. About 44% of respondents experienced a disruption in the regular contacts with their non- cohabitating children during the Covid outbreak.

**Table 2:** Descriptive Statistics.

Variables	Mean	SD	N
**Dependent Variable**			
Deepening depressed mood	0.151	0.358	15,508
Greater Anxiety	0.196	0.397	15,508
Greater Loneliness	0.114	0.318	15,508
Disruption of parent-child contacts	0.444	0.496	15,508
**Demographics**			
Age	74.01	6.525	15,508
Female	0.552	0.497	15,508
Married	0.665	0.471	15,508
**SES and Education**			
Low education level	0.318	0.466	15,508
Medium education level	0.431	0.495	15,508
High education level	0.249	0.432	15,508
Retired	0.886	0.317	15,508
Ends not meeting	0.276	0.447	15,508
**Health**			
# Chronic conditions	1.870	1.554	15,508
#Mobility limitations	1.567	2.146	15,508
**Cognitive Impairments**			
Episodic memory score	9.098	3.429	15,508
**Covid-19**			
Directly affected by Covid-19	0.014	0.120	15,508
Other people with Covid 19	0.09	0.096	15,508
Physical distance	0.946	0.225	15,508
**Contacts with others**			
Any contact with others	0.320	0.466	15,508
**Instruments**			
Non-cohabitation	0.883	0.320	15,508
Duration of stay-at-home restrictions (in weeks)	3.358	3.398	15,508
Internet Use	0.559	0.496	15,508

N.B: Author processing of data from SHARE wave 8 and SHARE Wave 8 COVID-19.

Table 3 shows marginal effects for the structural equation for a deepening depressed mood and the reduced form equation for the disruption of parent–adult child contacts.

**Table 3:** Results from the Recursive Bivariate Probit – Baseline Model.

	Disruption of parent-child relationship		Deepening depressed Mood	
Variables	Marginal effects	SE	Marginal effects	SE
Disruption of parent-child contacts	–	–	0.173 ***	0.052
**Demographics**				
Age	-0.004 ***	0.001	0.001	0.006
Female	0.010	0.008	0.081 ***	0.001
Married	-0.003	0.008	-0.013 **	0.006
**SES and Education**				
Medium education level	0.001	0.010	-0.001	0.007
High education level	0.034 ***	0.011	0.005	0.008
Retired	0.036 ***	0.013	-0.006	0.009
Ends not meeting	-0.003	0.010	0.052 ***	0.007
**Health**				
# chronic conditions	0.002	0.002	0.014 ***	0.002
#mobility limitations	0.002	0.002	0.009 ***	0.001
**Cognitive Impairments**				
episodic memory score	0.002	0.001	-0.002 **	0.001
**Covid-19**				
directly affected by Covid-19	0.042	0.032	0.058 ***	0.021
other people with Covid 19	-0.001	0.039	0.036	0.026
physical distance	0.074 ***	0.017	0.019	0.013
**Contacts with others**				
any contact with others	-0.057 ***	0.008	0.001	0.014
**Instruments**				
Non-cohabitation	0.154 ***	0.012	–	–
Duration of stay-at-home restrictions (in weeks)	0.015 ***	0.004	–	–
Internet use	0.054 ***	0.009		
**Country dummies**	yes		yes	
**Rho**			-0.436 ***	
N. obs	15,508		15,508	

N.B.: Sample Selection: individuals aged 65 + from Wave 8 and SHARE-Covid19 Survey.

Significance level: * p value < 0.1; ** p value < 0.05; *** p value < 0.01.

With specific reference to the reduced-form equation, our findings show that the binary indicators for non-cohabitating parent-children and the indicator for the duration of the lockdown restrictions (measured as the number of weeks since the beginning of the lockdown in each country in the study) are both strong predictors (significant at 99 %) of the disruption of parent-child contacts. As expected, during the Covid-19 pandemic, the likelihood of this disruption was higher (with an increase of about 15 %) with adult children who do not live with or close to their parents (i.e., in the same household or in the same building); older adults remained isolated in their homes with limited contacts with others, including those with non-cohabiting adult children, considered a critical factor in contributing to the spread of COVID-19 (Arpino et al., 2021, Bayer and Kuhn, 2020). The duration of movement restrictions and lockdowns also has a positive and significant effect on the disruption of parent-child contacts: an additional week of lockdown significantly increases the probability of this disruption by about 1.5 %.

Interestingly, the marginal effect of the indicator of regular Internet use is significant (again at 99 %) but positive. According to our results, regular Internet use increases the probability of the disruption of parent–adult child contacts by about 5.4 %. Arguably, as a new infectious disease, COVID-19 has drastically increased the sense of uncertainty among individuals. To learn more about the pandemic, people have relied heavily on the Internet, which has become one of the most popular sources of health information, affecting perceptions of risk and preventive behavior (Wang et al., 2020, Garfin et al., 2020). According to the recent literature, health information related to the Covid infection collected online has raised awareness of the disease and has been positively associated with engagement in all types of preventive behavior: not only wearing a face mask in public, hand washing, covering the nose and mouth when sneezing and coughing but also maintaining a distance from others and complying with stay-at-home orders, increasing the likelihood of disruptions to personal contacts especially among the elderly, who are vulnerable to Covid infections (Li et al., 2020).

With reference to the structural equation, our results show that a disruption in the contacts with offspring has positive and significant associations with mental health issues increasing the probability of suffering from worsened depressed mood by about 17 %. The emergence of COVID-19 and the measures implemented to curb its spread (such as physical distancing, stay-at-home orders, travel restrictions) have forced people indoors, reducing opportunities to remain socially connected, especially among older adults, at a higher risk of serious infection. Intergenerational family contacts, a significant part of overall relations and an important source of social and emotional support for older people, were also affected by these measures. During the COVID-19 pandemic, older people were asked or forced to reduce their physical contacts, included those with non-cohabiting adult children, considered a critical factor in contributing to the spread of COVID-19 (Arpino et al., 2021, Bayer and Kuhn, 2020). In European countries, however, welfare systems rely heavily on intergenerational solidarity, and the informal care of the elderly is carried out overwhelmingly by (non-cohabiting) adult offspring who provide assistance to their parents with their routine day-to-day activities. The containment measures adopted by almost all European countries have often led to a disruption of interpersonal contacts between older parents and their children, creating a new set of challenges including mental health consequences.

Among other factors affecting the probability of a deterioration in mental health, our results show a significant effect for the worsening economic situation of respondents. Making ends meet with great or some difficulty significantly increases the likelihood of elderly respondents feeling sadder or depressed. Moreover, reporting higher scores in the episodic memory test is negatively associated with the probability of worsening mental health. This finding confirms that poorer cognitive functioning is related to greater symptoms of depression (Perrino et al., 2008). Finally, married individuals are less likely to report being sad or depressed during the outbreak, compared to the elderly who live alone. Other factors that negatively influence mental health are gender (female), suffering from mobility limitations, multiple chronic conditions and being directly affected by Covid-19.

As mentioned above, we estimated the two equations for the probability of a disruption in parent-children contacts and mental health issues, using the recursive bivariate probit specification. This allowed us to test for unobserved heterogeneity, the effect of which was captured by the correlation between the error terms from the single equation models. By simultaneously estimating the two equations and considering the correlation in the error terms, we controlled for the effect of unobserved factors. Table 3, Table 4 show the correlation for the full recursive models. The null hypothesis of exogeneity was rejected in both cases.

**Table 4:** Results from the Recursive Bivariate Probit with a different threshold for the disruption of parent-child contacts.

	Disruption of parent-child relationship		Deepening depressed Mood	
Variables	Marginal effects	SE	Marginal effects	SE
Disruption of parent-child contacts	–	–	0.187 ***	0.052
**Demographics**				
Age	-0.005 ***	0.001	0.001	0.001
Female	0.003	0.008	0.081 ***	0.006
Married	0.001	0.008	-0.014 **	0.006
**SES and Education**				
Medium education	-0.008	0.009	0.001	0.007
High education	0.019 *	0.011	0.009	0.008
Retired	0.027 **	0.012	-0.004	0.009
Ends not meeting	0.010	0.010	0.049 ***	0.007
**Health**				
# chronic conditions	-0.002	0.002	0.015 ***	0.001
Mobility limitations	0.002	0.002	0.009 ***	0.001
**Cognitive Impairments**				
episodic memory score	0.001	0.001	-0.002 **	0.001
**Covid-19**				
directly affected by Covid-19	0.020	0.031	0.062 ***	0.021
other people with Covid 19	0.014	0.038	0.033	0.026
physical distance	0.059 ***	0.017	0.022	0.013
**Contacts with others**				
any contact with others	-0.048 ***	0.008	0.001	0.006
**Instruments**				
Non-cohabitation	0.121 ***	0.012	–	–
Duration of stay-at-home restrictions (in weeks)	0.016 ***	0.004	–	–
Use of internet	0.032 ***	0.009		
**Country dummies**	yes		yes	
**Rho**			-0.472 ***	
N. obs	15,508		15,508	

N.B: Sample Selection: individuals aged 65 + from Wave 8 and SHARE-Covid19 Survey.

Significance level: * p value < 0.1; ** p value < 0.05; *** p value < 0.01.

The correlation parameter between the disruption of parent-child contacts and depression indicates whether and how unobservable factors jointly affect the disruption and the mental health of parents. Our results indicate a negative statistically significant correlation between the disturbance of the two equations i.e., unobservable variables that increase the likelihood of depression decrease the probability of disruption in parent-children contacts.^8^

### 4.1 Sensitivity checks

As a sensitivity check, we re-ran the model, slightly changing the dummy variable measuring the likelihood of disruption of “regular contacts” with children compared to the baseline model. As stated above, the variable was constructed comparing the respondents’ answers to questions about the frequency of in-person and electronic contacts included in the social network module of the 8th wave of SHARE with those reported in the SHARE Corona Survey. We assessed the frequency of respondents’ contacts with children who do not live in the same household based on the options provided in SHARE which range from (i) daily; (ii) several times a week; (iii) about once a week; (iv) about every two weeks; about once a month; (v) less than once a month; (vi) never. In the baseline model, “regular contacts” were contacts in the range from “daily” to “about once a week”. We constructed a binary variable with value one if those who reported having regular contacts with children before the outbreak also reported contacts with them “less often” or “never” during the outbreak. As a sensitivity check, “regular contacts” were defined, using a different threshold ranging from “daily” to “several times a week”. According to the existing literature, high contact frequency can be considered a proxy indicator of strong family ties and potential support for older people (Worsfeld, 2011). The results are shown in Table 4.

Table 4 shows that the results are consistent with those obtained from the baseline model. Interestingly, the marginal effect of the disruption of parent-child contacts remains highly significant (at 99 %) but greater than in the baseline model, increasing from 0.173 to 0.187. Our findings revealed that the higher the frequency of contacts between parents and children before the Covid outbreak, the greater the impact of the disruption on the psychological well-being of the elderly parents.^9^

As a further sensitivity analysis, the baseline model was also tested considering two other symptoms of mental health: greater anxiety and greater loneliness. We considered anxiety since, like a depressed mood, it is a key symptom of mental health disorder (see Kalin, 2020). Moreover, we also considered feeling lonely. Indeed, previous research has recognized that perceived social isolation and loneliness are major risk factors for mental health problems, including depression and generalized anxiety, especially in later life (Ong et al., 2016, Bu et al., 2020). Feeling lonely in particular has increased during the pandemic, especially among older people, due to mobility restrictions, social isolation and lockdown measures introduced to tackle the spread of the Coronavirus (Santini et al., 2020, Santini et al., 2020).

In relation to anxiety, respondents were asked the following questions: “In the last month, have you felt nervous, anxious, or on edge?”, with yes or no answer options. If the answer was “yes”, respondents were also asked “Has that been more so, less so, or about the same as before the outbreak of Corona?”. Based on their answers, it was possible to create a dummy indicator that captures greater anxiety symptoms, considered to be the case if respondents reported “more so” and not the case otherwise (“less so or about the same”).

For loneliness, respondents were asked “How much of the time do you feel lonely?”, with response options being often, some of the time, hardly ever or never. Greater loneliness was assessed among those responding “often or some of the time” to the first question, and they were also asked “Has that been more so, less so, or about the same as before the outbreak of Corona?”. We constructed a binary variable with value one if they reported “more so” and zero otherwise (“less so or about the same”). Again, the outcomes described above are all measures of the worsening of mental health during the COVID-19 outbreak and are not simply indicators of the existence or absence of symptoms.^10^

Table 5, Table 6 show that the results remained robust under different specifications of the dependent variable.

**Table 5:** Results from the Recursive Bivariate Probit, Greater Anxiety.

	Disruption of parent-child relationship		Greater Anxiety	
Variables	Marginal effects	SE	Marginal effects	SE
Dispruption of parent-child contacts	–	–	0.154 ***	0.053
**Demographics**				
age	-0.004 ***	0.001	-0.001	0.001
female	0.010	0.008	0.083 ***	0.006
married	-0.003	0.008	0.015 **	0.007
**SES and Education**				
Medium education level	0.001	0.010	0.015 **	0.007
High education level	0.035 ***	0.011	0.025 ***	0.009
Retired	0.036 ***	0.013	0.001	0.010
Ends not meeting	-0.002	0.010	0.040 ***	0.008
**Health**				
# chronic conditions	0.002	0.002	0.014 ***	0.002
#mobility limitations	0.002	0.002	0.010 ***	0.001
**Cognitive Impairments**				
episodic memory score	0.002	0.001	-0.003 ***	0.001
**Covid-19**				
directly affected by Covid-19	0.042	0.032	0.060 **	0.023
other people with Covid 19	-0.003	0.040	0.079 ***	0.028
physical distance	0.074 ***	0.017	0.040 ***	0.015
**Contacts with others**				
any contact with others	-0.057 ***	0.008	0.007	0.007
**Instruments**				
Non-cohabitation	0.156 ***	0.012	–	–
Duration of stay-at-home restrictions (in weeks)	0.015 ***	0.004	–	–
Internet use	0.053 ***	0.009		
**Country dummies**	yes		yes	
**Rho**			-0.320 **	
N. obs	15508		15508	

N.B: Sample Selection: individuals aged 65 + from Wave 8 and SHARE-Covid19 Survey.

Significance level: * p value < 0.1; ** p value < 0.05; *** p value < 0.01.

**Table 6:** Results from the Recursive Bivariate Probit, Greater Loneliness.

	Disruption of parent-child relationship		Greater Loneliness	
Variables	Marginal effects	SE	Marginal effects	SE
parent-children contact disruption	–	–		
**Demographics**			0.167 ***	0.038
age	-0.004 ***	0.001	0.001 ***	0.001
female	0.010	0.008	0.060 ***	0.005
married	-0.003	0.008	-0.072	0.005
**SES and Education**				
Medium education	0.001	0.010	0.001	0.006
High education	0.034 ***	0.011	0.005	0.007
Retired	0.036 ***	0.013	0.015 *	0.008
Ends not meeting	-0.003	0.010	0.040 ***	0.006
**Health**				
# chronic conditions	0.002	0.002	0.005 ***	0.001
#mobility limitations	0.002	0.002	0.007 ***	0.001
**Cognitive Impairments**				
episodic memory score	0.001	0.001	-0.002 ***	0.001
**Covid-19**				
directly affected by Covid-19	0.042	0.031	0.024	0.020
other people with Covid 19	-0.002	0.040	0.013	0.025
physical distance	0.074 ***	0.017	0.023 *	0.012
**Contacts with others**				
any contact with others	-0.058 ***	0.008	0.001	0.006
**Instruments**				
Non-cohabitation	0.156 ***	0.012	–	–
Duration of stay-at-home restrictions (in weeks)	0.015 ***	0.004	–	–
Internet use	0.058 ***	0.009		
**Country dummies**	yes		yes	
**Rho**			-0.473 ***	
N. obs	15,508		15,508	

N.B: Sample Selection: individuals aged 65 + from Wave 8 and SHARE-Covid19 Survey.

Significance level: * p value < 0.1; ** p value < 0.05; *** p value < 0.01.

The marginal effect of the disruption of parent-children contacts remains highly significant (once again at 99 %). The factors that significantly influence greater anxiety and loneliness again include the worsening economic situation of respondents, their gender (female), suffering from mobility limitations, multiple chronic conditions and being directly affected by Covid-19. The probability of suffering from greater loneliness in particular also increases with age.^11^

Finally, we test whether the marginal effect of the disruption of parent-children contacts on a deepening depressed mood varies by gender, age groups and respondents’ education. Specifically, concerning age groups we considered first people aged 70 and over and then people aged 75 s and over; for respondents’ education we re-run the model dividing the sample into two groups: poorly educated against medium and highly educated. These analyses show that the estimated marginal effect is present in all groups of individuals considered: the disruption of parent–child contacts due COVID-19 pandemic and related lockdown policies appears to be a significant element for understanding the deterioration of mental health among older Europeans in times of COVID-19, irrespective of gender, age and level of education. Our results, included in Table 7, Table 8, Table 9 show that the sign of the estimated marginal effects is always positive and statistically significant. Our findings suggest that especially men, older people (aged 70 and over), as well as individuals with a lower level of education are at higher risk for mental health problems related to disrupted personal parent–child contacts during this challenging time.

**Table 7:** Results from the Recursive Bivariate Probit by gender – Baseline Model for Deepening depressed Mood.

	Males		Females	
	Disruption of parent-child relationship	Deepening depressed Mood	Disruption of parent-child relationship	Deepening depressed Mood
Variables	Marginal effects	Marginal effects	Marginal effects	Marginal effects
Disruption of parent-child contacts	–	0.196 ***	–	0.149 **
		(0.076)		(0.066)
**Demographics**				
Age	-0.003 * **	0.001	-0.005 ***	0.001
	(0.001)	(0.001)	(0.001)	(0.001)
Married	-0.027 *	-0.022 **	0.005	-0.010
	(0.014)	(0.009)	(0.011)	(0.008)
**SES and Education**				
Medium education level	-0.008	-0.028 ***	0.008	0.019 *
	(0.015)	(0.010)	(0.013)	(0.010)
High education level	0.012	-0.018	0.057 ***	0.028 **
	(0.017)	(0.011)	(0.016)	(0.013)
Retired	0.050 **	-0.017	0.027	-0.003
	(0.023)	(0.016)	(0.016)	(0.013)
Ends not meeting	-0.005	0.053 ***	-0.001	0.053 ***
	(0.016)	(0.010)	(0.013)	(0.010)
**Health**				
# chronic conditions	0.006	0.012 ***	0.001	0.017 ***
	(0.004)	(0.002)	(0.003)	(0.002)
# mobility limitations	0.004	0.009 ***	0.001	0.010 ***
	(0.003)	(0.002)	(0.002)	(0.002)
**Cognitive Impairments**				
episodic memory score	-0.003 *	-0.001	0.001	-0.003 **
	(0.002)	(0.001)	(0.001)	(0.001)
**Covid-19**				
directly affected Covid-19	-0.006	0.086 ***	0.080 *	0.028
	(0.046)	(0.026)	(0.044)	(0.033)
other people with Covid 19	0.076	0.065 **	-0.072	-0.001
	(0.056)	(0.032)	(0.056)	(0.042)
Physical distance	0.054 **	-0.020	0.094 ***	0.070 * **
	(0.024)	(0.016)	(0.025)	(0.002)
**Contacts with others**				
any contact with others	-0.052 ***	0.003	-0.060 ***	0.001
	(0.013)	(0.009)	(0.011)	(0.009)
**Instruments**				
Non-cohabitation	0.145 ***	–	0.160 ***	–
	(0.020)		(0.024)	
Duration of stay-at-home restrictions (in weeks)	0.015 **	–	0.016 ***	–
	(0.006)		(0.005)	
	(0.006)		(0.004)	
Internet use	0.057 ***	–	0.052 ***	–
	(0.014)		(0.012)	
**Country dummies**	yes	yes	yes	yes
**Rho**		-0.586 ***		-0.318 *
N. obs	6942	6942	8566	8566

N.B: Sample Selection: Males and females aged 65 + from Wave 8 and SHARE-Covid19 Survey. Significance level: * p value < 0.1; ** p value < 0.05; * ** p value < 0.01.

**Table 8:** Results from the Recursive Bivariate Probit by age-group - Baseline Model for Deepening depressed Mood.

	Age > =70		Age > = 75	
	Disruption of parent-child relationship	Deepening depressed mood	Disruption of parent-child relationship	Deepening depressed mood
Variables	Marginal effects	Marginal effects	Marginal effects	Marginal effects
Disruption of parent-child contacts	–	0.222 ***	–	0.221 ***
		(0.049)		(0.067)
**Demographics**				
Age	-0.006 ***	0.001 **	-0.008 ***	0.002
	(0.001)	(0.001)	(0.001)	(0.001)
female	-0.002	0.085 ***	-0.010	0.086 ***
	(0.010)	(0.007)	(0.013)	(0.010)
married	0.001	-0.006	0.008	-0.002
	(0.010)	(0.007)	(0.013)	(0.010)
**SES and Education**				
Medium education level	-0.003	-0.004	0.002	-0.016
	(0.014)	(0.008)	(0.015)	(0.011)
High education level	0.031 **	0.002	0.018	0.007
	(0.014)	(0.010)	(0.018)	(0.013)
Retired	0.027	0.003	0.009	0.009
	(0.018)	(0.013)	(0.025)	(0.018)
Ends not meeting	0.001	0.052 ***	-0.010	0.067 ***
	(0.012)	(0.009)	(0.016)	(0.011)
**Health**				
# chronic conditions	-0.001	0.013 ***	-0.007 *	0.014 ***
	(0.003)	(0.002)	(0.004)	(0.003)
# mobility limitations	0.002	0.009 ***	0.001	0.008 ***
	(0.002)	(0.001)	(0.002)	(0.002)
**Cognitive Impairments**				
episodic memory score	0.001	-0.002 **	0.001	-0.001
	(0.001)	(0.001)	(0.002)	(0.001)
**Covid-19**				
directly affected Covid-19	0.026	0.076 ***	0.009	0.087 **
	(0.040)	(0.027)	(0.053)	(0.037)
other people with Covid 19	-0.016	0.037	-0.009	0.051
	(0.050)	(0.033)	(0.065)	(0.044)
Physical distance	0.080 ***	0.009	0.088 ***	0.011
	(0.020)	(0.016)	(0.024)	(0.019)
**Contacts with others**				
any contact with others	-0.054 ***	0.03	-0.038 ***	-0.011
	(0.010)	(0.008)	(0.013)	(0.010)
**Instruments**				
Non-cohabitation	0.162 * **	–	0.162 ***	
	(0.015)		(0.018)	
Duration of stay-at-home restrictions (in weeks)	0.014 * **		0.013 *	
	(0.005)		(0.007)	
Internet use	0.058 * **		0.047 ***	
	(0.011)		(0.014)	
**Country dummies**	yes	yes		
**Rho**		-0.563 * **		-0.563 **
N. obs	10888	10888	6453	6453

N.B: Sample Selection: Individuals aged 70 + and 75 + from Wave 8 and SHARE-Covid19 Survey. Significance level: * p value < 0.1; ** p value < 0.05; *** p value < 0.01.

**Table 9:** Results from the Recursive Bivariate Probit by education - Baseline Model for Deepening depressed Mood.

	Low Education level		Medium/High education level	
	Disruption of parent-child relationship	Deepening Depressed Mood	Disruption of parent-child relationship	Deepening depressed Mood
Variables	Marginal effects	Marginal effects	Marginal effects	Marginal effects
Disruption of parent-child contacts	–	0.183 **	–	0.143 **
		(0.089)		(0.061)
**Demographics**				
age	-0.004 ***	0.001	-0.004 ***	0.001
	(0.001)	(0.009)	(0.001)	(0.001)
female	-0.005	0.052 ***	0.017 *	0.092 ***
	(0.015)	(0.012)	(0.010)	(0.007)
married	0.008	-0.015	-0.008	-0.012 *
	(0.015)	(0.011)	(0.010)	(0.007)
**SES and Education**				
Retired	0.026	-0.005	0.035 **	-0.012
	(0.021)	(0.016)	(0.016)	(0.011)
Ends not meeting	-0.010	0.069 ***	-0.004	0.043 ***
	(0.017)	(0.013)	(0.013)	(0.008)
**Health**				
# chronic conditions	0.001	0.017 ***	0.003	0.012 ***
	(0.004)	(0.003)	(0.003)	(0.002)
# mobility limitations	-0.001	0.014 ***	0.004	0.007 ***
	(0.003)	(0.002)	(0.002)	(0.001)
**Cognitive Impairments**				
episodic memory score	0.001	-0.002	0.002	-0.002 *
	(0.002)	(0.001)	(0.001)	(0.001)
**Covid-19**				
directly affected Covid-19	0.067	0.082 *	0.033	0.052 **
	(0.062)	(0.045)	(0.037)	(0.023)
other people with Covid 19	-0.055	-0.005	0.031	0.064 **
	(0.062)	(0.046)	(0.052)	(0.032)
Physical distance	0.082 * **	0.019	0.072 ***	0.019
	(0.028)	(0.024)	(0.022)	(0.017)
**Contacts with others**				
any contact with others	-0.053 * **	-0.004	-0.058 ***	0.004
	(0.015)	(0.012)	(0.010)	(0.008)
**Instruments**				
Non-cohabitation	0.099 * **	–	0.191 ***	–
	(0.019)		(0.016)	
Duration of stay-at-home restrictions (in weeks)	0.021 * *	–	0.019 ***	–
	(0.008)		(0.005)	
Internet use	0.058 * **	–	0.060 ***	–
	(0.016)		(0.011)	
**Country dummies**	yes	yes	yes	yes
**Rho**		-0.423 *		-0.379 **
N. obs	4946	4946	10562	10562

N.B: Sample Selection: Individuals aged 65 + from Wave 8 and SHARE-Covid19 Survey. Significance level: * p value < 0.1; ** p value < 0.05; *** p value < 0.01.

## 5 Conclusions

The COVID-19 pandemic and the lockdown measures adopted in almost all European countries to cope with the spread of coronavirus have drastically affected the daily life of the whole population. Older people, from the outset of the COVID crises are identified as the most vulnerable portion of the population, have been faced with unique challenges in arranging how to manage their health and care needs without leaving home. Indeed, the social distancing measures necessary to protect them against the risk of serious infection, have in many cases also meant the disruption of social contacts and contacts with their family, in particular with their adult children, their primary source of informal care and social support.

Using the 8th wave of the SHARE and the SHARE Corona Survey, we investigated whether the disruption of parent–adult child contacts due to social distancing restrictions increased the symptoms of depression in the elderly during the first wave of the COVID-19 pandemic. We constructed a joint model of parent-child contact disruption and mental health issues, estimated by using a recursive bivariate probit model taking into account unobserved heterogeneity between individuals, as may characterize this relationship.

Our evidence reflected that home confinement due to COVID-19 leading to a disruption of interpersonal contacts between older parents and children, may indeed have created a new set of challenges including mental health consequences, all the tougher the stronger the family ties. Therefore, policy interventions should take into account the fact that interpersonal contacts, especially with family members and adult children, are not just a potential vehicle of transmission of the virus, but are also a source of support which may help to counterbalance the negative consequences on mental health of policy responses to the outbreak.

## Funding

None.

## Authors’ contributions

The interpretation and reporting of the results are the sole responsibility of the authors. All authors contributed to the conception of the study, the design of the study, and writing of the manuscript. All the authors commented on/edited all drafts of the manuscript. All the authors can be identified as the guarantors for the overall content and interpretation of the results.

## Conflict of interest

The authors report no declarations of interest.

## Declaration of Competing Interest

The authors declare no competing interests. The views expressed are purely those of the authors and may not in any circumstances be regarded as stating any official position of the European Commission.

## Figures and Tables

**Table 1A:** Results from the Recursive Bivariate Probit without exclusion restrictions (Wilde).

	Disruption of parent-child relationship equation		Deepening depressed Mood	
Variables	Marginal effects	SE	Marginal effects	SE
Disruption of parent-child contacts	–	–	0.200 ***	0.077
**Demographics**				
Age	-0.004 ***	0.006	0.001	0.001
Female	0.010	0.008	0.080 ***	0.006
Married	-0.003	0.008	-0.013 **	0.006
**SES and Education**				
Medium education	0.001	0.010	-0.001	0.007
High education	0.034 ***	0.011	0.006	0.009
Retired	0.036 ***	0.013	-0.007	0.009
Ends not meeting	-0.002	0.010	0.051 ***	0.007
**Health**				
# chronic conditions	0.002	0.002	0.014 ***	0.002
#mobility limitations	0.002	0.002	0.009 ***	0.001
**Cognitive Impairments**				
episodic memory score	0.001	0.001	-0.002 **	0.001
**Covid-19**				
directly affected by Covid-19	0.041	0.032	0.057 ***	0.021
other people with Covid 19	0.001	0.039	0.036	0.026
physical distance	0.074 ***	0.017	0.018	0.015
**Contacts with others**				
any contact with others	-0.057 ****	0.008	0.004	0.008
**Instruments**				
Non-cohabitation	0.153 ***	0.020	-0.001	0.023
Duration of stay-at-home restrictions (in weeks)	0.015 ***	0.005	-0.003	0.004
Internet use	0.056 ***	0.009	-0.007	0.008
**Country dummies**	Yes		yes	
**Rho**			-0.499 **	
N. obs	15508		15508	

N.B: Sample Selection: individuals aged 65 + from Wave 8 and SHARE-Covid19 Survey.

Significance level: * p value < 0.1; ** p value < 0.05; *** p value < 0.01.

**Table 2A:** Type and Duration of Restrictions During the First Wave of COVID-19 Pandemic.

Country	Lockdown		Restrictions	No. of weeks in Lockdown
	start	end		
Germany	–	–	Non-essential shops closed, ban on gatherings, stay-at-home-advice to general population	0
Sweden	–	–	Ban on gatherings, stay-at-home advice to at-risk population	0
Netherlands	–	–	Non-essential shops closed, ban on gatherings, stay-at-home-advice to general population	0
Spain	14/03/2020	11/5/2020	Stay-at-home-order	8.28
Italy	10/3/2020	4/5/2020	Stay-at-home-order	7.85
France	17/03/2020	11/5/2020	Stay-at-home-order	7.85
Denmark	–	–	Non-essential shops closed, ban on gatherings, stay-at-home-advice to at-risk population	0
Greece	23/03/2020	4/5/2020	Stay-at-home-order	6
Switzerland	–	–	Non-essential shops closed, ban on gatherings, stay-at-home-advice to at-risk population	0
Belgium	18/03/2020	9/5/2020	Stay-at-home order	7.42
Israel	19/03/2020	4/5/2020	Stay-at-home order	6.57
Czech Republic	16/03/2020	24/04/2020	Stay-at-home order	5.57
Poland	24/03/2020	18/04/2020	Stay-at-home order	3.57
Luxembourg	18/03/2020	19/04/2020	Stay-at-home order	4.57
Hungary	27/03/2020	18/05/2020	Stay-at-home order	7.42
Slovenia	19/03/2020	4/5/2020	Stay-at-home order	6.57
Estonia	–	–	Non-essential shops closed, ban on gatherings, stay-at-home-advice to general population	0
Croatia	–	–	Non-essential shops closed, ban on gatherings, stay-at-home-advice to general population	0
Lithuania			Non-essential shops closed, ban on gatherings, stay-at-home-advice to general population	0
Bulgaria	–	–	Non-essential shops closed, ban on gatherings	0
Cyprus	24/03/2020	3/5/2020	Stay-at-home order	5.71
Finland	–	–	Ban on gatherings, stay-at-home advice to at-risk population	0
Latvia	–	–	Ban on gatherings, stay-at-home advice to general population	0
Malta	–	–	Non-essential shops closed, ban on gatherings, stay-at-home advice to at-risk population	0
Romania	22/03/2020	13/05/2020	Stay-at-home order	7.42
Slovakia	–	–	Non-essential shops closed, ban on gatherings	0

This Table summarizes the type and duration of the restrictions adopted in various European countries during the first wave of the COVID-19 pandemic.

Source: European Centre for Disease Prevention and Control website (https://www.ecdc.europa.eu/en/publications-data/download-data-response-measures-covid-19)

N.B.: Lockdown is considered as the result of a "Stay-at-home order" for the general population.

A "Stay-at-home order" is an order from a government authority that severely restricts the movement of the population, adopted as a mass quarantine strategy.

**Table 3A:** Univariate Probit Model.

	Univariate Probit	
**Dependent Variable**: Deepening depressed mood		
Variables	Margins	SE
parent-children contact disruption	0.010 *	0.005
**Demographics**		
age	-0.001	0.001
female	0.081 ***	0.006
married	-0.012 **	0.006
**SES and Education**		
Medium education	0.001	0.007
High education	0.015 *	0.008
Retired	0.001	0.009
Ends not meeting	0.051 ***	0.007
**Health**		
# chronic conditions	0.014 ***	0.001
#mobility limitations	0.009 ***	0.001
**Cognitive Impairments**		
episodic memory score	-0.001 **	0.001
**Covid-19**		
directly affected by Covid-19	0.066 ***	0.020
other people with Covid 19	0.035	0.026
physical distance	0.031 **	0.013
**Contacts with others**		
any contact with others	-0.006	0.006
Country dummies	yes	
N. obs	15508	

Significance level: * p value < 0.1; ** p value < 0.05; *** p value < 0.01.

**Table 4A:** Correlation Matrix Outcome Variables.

		Worsened Depressed Mood	Worsened Anxiety	Worsened Loneliness
Worsened Depressed Mood		1.0000		
Greater Anxiety		0.4763	1.0000	
Greater Loneliness		0.3413	0.2587	1.0000

## Appendix

1. A Wilde's (2000) Identification Approach (without exclusion restrictions).

For our estimation we employed a recursive bivariate probit model since we assumed that the disruption of parent–child contacts due to COVID-19 and the probability of suffering from a deepening depressed mood were determined simultaneously since we cannot exclude a priori that common unobservable enter both equations or that the unobservable of one equation are correlated with those of the other. Conventionally, the identification of a recursive bivariate probit model has been based on exclusion restrictions to obtain a more robust identification of the parameters. Maddala (1983, p. 123) suggests that at least one of the reduced-form exogenous variables be not included in the structural equations as explanatory variables. However, more recent work by Wilde (2000) demonstrated that identification is achieved even if the same regressors appear in all the equations provided each equation contains at least one varying exogenous regressor.

Following Wilde’s (2000) approach we run a sensitivity analysis to compare a model with no exclusion restrictions, where each equation has the same regressor matrix, to the model with the identifying assumption. The results of this sensitivity analysis are shown in Table 1A. We find only a marginal variation in the coefficients moving from one specification to another; hence the results are robust to this different identification assumption.

It is worth to notice that the variables that we employed as exogenous instruments in the model with exclusion restrictions (Maddala, 1983) do not significantly predict the probability of suffering from a deepening depressed mood.

See Table 1A.

## Footnotes

1 The outcome described is a measure of the deterioration in mental health conditions during the COVID-19 outbreak and is not simply an indicator of the existence or absence of a symptom of mental distress; this measure can take into account the higher risk of being negatively affected by the disruption in parent-child contacts for older individuals with depressed mood symptoms before the pandemic.

2 Respondents were asked about their depressed mood in the month before the interview (with yes or no answers) and whether symptoms have worsened, improved, or remained the same since the beginning of the pandemic. The answers cannot be scored simply (for example as 1, 2, 3) because the true scale would not be equidistant between categories (O’Donnell et al., 2008). Hence, according to previous literature (see for instance García-Prado et al., 2022), we constructed a dummy indicator that captures worsened depressed mood, considered present if respondents reported “more so” and absent otherwise (“less so or about the same”). This practice avoids the imposition of a scale although some information is lost (Wagstaff and Van Doorslaer, 1994).

3 The first lockdown in spring 2020 drastically changed communications pattern: people were required to reduce their face-to-face contacts and digital communication became the “new norm” for social interaction. By helping individuals to cope with the difficulties of home confinement, digital communication has partially buffered against the negative impact of social restrictions reducing the feeling of loneliness and isolation (Canale et al., 2020, Gabbiadini et al., 2020). According to the previous literature people shifted their communication to instant messengers, video calls and interaction on social media (Meier et al., 2021), replacing physical contacts (e.g., with children) during the COVID-19 pandemic (Hajek, and König, 2021). While the SHARE Covid Survey enables different forms of contact with social network members to be distinguished—e.g., in-person, by phone or mail, email or any other electronic means, the 8th wave of SHARE does not. Hence, it was not possible to identify a substitution effect i.e., whether the reduction of in-person contacts during the first wave of the COVID-19 pandemic was replaced by electronic contacts.

4 The STATA software provides the statistic z = $\hat{\rho }/S_{\hat{\rho }}\;$to test the hypothesis H_0_: $\rho_{\mathrm{jk}}=0$. If the error terms are independent, the maximum simulated likelihood estimation is equivalent to the separate maximum likelihood probit estimation.

5 We find only a marginal variation in the coefficients moving from one specification to another; hence the results are robust to this different identification assumption.

6 Lockdown policies differed from country to country, from a soft recommendation to stay at home to a moderate stay at home directive or an order requiring everyone to be home except for essential purposes. In our sample, the countries that introduced “stay-at-home orders” are: Belgium, France, Italy, Romania, Greece, Hungary, Luxembourg, Poland, Spain, Slovenia, Cyprus, Czech Republic, and Israel (see Table 2A in the Appendix). Country-level information on lockdowns was drawn from the European Centre for Disease Prevention and Control (https://www.ecdc.europa.eu/en/publications-data/download-data-response-measures-covid-19).

7 Concerning exclusion restrictions, to balance the statistical fit of the model and to ensure sufficiently parsimonious parametrization, we used Bayesian information criterion, as proposed by Schwarz (1978). Using this criterion involves excluding from the structural equation the variables that describe the geographical proximity of aging parents and their children and the duration of the lockdown policy measured as the number of weeks since the beginning of the lockdown in each country in the study. These variables were assumed to affect a deepening depressive mood indirectly through the disruption of the parent-child relationship. The Bayesian information criterion also involves excluding from the structural equation the variable that measures regular Internet use, included to proxy older users’ ability to harness the Internet to cope with pandemic-induced social network disruption.

8 The statistically significant correlation coefficients suggest that the hypothesis of independence across the error terms of the two latent equations can be rejected, and the bivariate probit model is better for the observed data. Table 3A in the Appendix contains marginal effects for the parent-child contacts disruption dummy on the probability of reporting worsened depressive mood when using a univariate probit model that does not allow for correlation between the errors of the mental health and the parent-child contacts disruption equations and hence do not control for endogeneity. A disruption in the contacts with offspring has still a positive association with mental health issues but the effect is not as strong as in the bivariate probilt model and is significant at 10 % only.

9 According to the previous literature strong family ties are negatively correlated with individuals generalized trust (Ermisch and Gambetta, 2010) and a higher intensity of the family relationship may also reduce “the capacity of the family members to interact in the outside world” (Herreros, 2015). Arguably, this may be the explanation why the effect of the parent-child contacts disruption on the psychological well-being of the elderly parents tend to be greater when the frequency of contact before the COVID-19 outbreak were higher (and family ties were stronger).

10 Table 4A in the Appendix shows the correlation matrix for the three self-reported psychological worsening distress symptoms, namely a deepening depressed mood, greater anxiety and greater loneliness.

11 We also re-run the model by replacing the dependent variable (a deepening depressed mood) with the presence of the depressed mood symptom and controlling for the presence of the depressed mood symptom before the COVID-19 pandemic (this information was collected from the 8th wave of SHARE). The results of this additional robustness exercise were in line with the baseline model. For the sake of brevity these results were not included in the paper but are available on request from the authors.
